# GPD1 deficiency—a rare, overlooked cause of liver disease

**DOI:** 10.1038/s10038-025-01339-9

**Published:** 2025-04-11

**Authors:** Necati Emrecan Türk, Serkan Belkaya, Selçuk Teke, Ceyda Tuna Kırsaçlıoğlu, Fatma Tuba Eminoğlu, Tunahan Çalıkoğlu, Aydan Kansu, Zarife Kuloglu

**Affiliations:** 1 Ankara University, School of Medicine, Department of Pediatrics, Division of Gastroenterology, Hepatology and Nutrition, Ankara, Turkey; 2 Bilkent University, Faculty of Science, Department of Molecular Biology and Genetics, Ankara, Turkey; 3 Ankara University, School of Medicine, Department of Pediatrics, Division of Metabolism, Ankara, Turkey

**Keywords:** Non-alcoholic steatohepatitis, Non-alcoholic fatty liver disease

## Abstract

Transient infantile hypertriglyceridemia is one of the diseases that should be considered in case of unexplained elevated liver enzymes, hypertriglyceridemia and hepatosteatosis. We report 2 siblings with novel homozygous variants in the GPD1 gene with transient infantile hypertriglyceridemia. Two siblings born from consanguineous marriage were referred due to hepatomegaly, elevated transaminases and fatty liver. After excluding other possible causes of fatty liver and elevated transaminase levels; whole-exome sequencing (WES) was performed on genomic DNA isolated from the peripheral blood samples of both patients. Whole exome sequencing revealed the identification of a novel homozygous variant, c.628 G > C:p.G210R, in GPD1. Our report underscores the importance of genome sequencing in diagnosing unexplained childhood fatty liver disease and/or elevated enzyme levels. In patients with transient infantile hypertriglyceridemia, investigation into novel homozygous variants in the GPD1 gene should be conducted using whole exome sequencing.

## Introduction

Transient infantile hypertriglyceridemia (HTGTI) is an uncommon genetic disorder characterized by hepatomegaly, temporary elevation of triglyceride levels, heightened levels of liver enzymes, enduring fatty liver, and hepatic fibrosis, typically appearing during early infancy. Mutations in the cytosolic isoform of glycerol-phosphate dehydrogenase (GPD) encoded by the GPD1 gene located on chromosome 2q12-q13 lead to this disease. Glycerol-3-phosphate dehydrogenase (GPD1) is a NAD-dependent (nicotinamide adenine dinucleotide) enzyme. This enzyme facilitates the reversible redox reaction between glycerol-3-phosphate and NAD, as well as between dihydroxyacetone phosphate (DHAP) and reduced NAD (NADH) [1]. It plays a crucial role in integrating carbohydrate and lipid metabolism by participating in the Embden-Meyerhof glycolysis pathway, gluconeogenesis, as well as triglyceride synthesis and degradation. Herein, we report 2 siblings with a novel homozygous variant of the GPD1 gene, characterized by elevated liver enzymes, hypertriglyceridemia, and hepatosteatosis.

## Case presentation

### Case-1

An 8-year-old girl presented at our hospital for further evaluation owing to persistent steatohepatitis. The patient was born from a first-degree cousin marriage at the 42nd week with a birth weight of 4200 g. She suffered from an afebrile seizure at 18 months of age and was mechanically ventilated for 15 days due to respiratory failure. During this period, hepatosplenomegaly and elevated liver enzymes were noticed. Abdominal ultrasonography (USG) showed hepatosteatosis. Her brother and mother were β-thalassemia carriers. One of her maternal aunts was suffering from hepatosteatosis and one of her paternal aunts was suffering from hepatosplenomegaly and hepatosteatosis too. On admission, her weight and length were normal. She had hepatomegaly (5 cm). In laboratory tests, her transaminase levels and hepatic function tests showed results within the normal range. Serum lipid tests revealed elevated triglyceride (353 mg/dL; normal range (n.r.): <150 mg/dL) and total cholesterol levels (216 mg/dL, n.r: <200 mg/dL). Abdominal USG revealed borderline hepatosplenomegaly and grade 1 hepatosteatosis. Mild elevation in transaminase levels was observed during follow-up. Additional investigations to explore potential differentials of steatohepatitis with hypertriglyceridemia, including infectious diseases, Wilson’s disease, autoimmune hepatitis, alpha-1 antitrypsin deficiency, thyroid dysfunction, celiac disease, hemochromatosis, and other possible metabolic disorders showed normal results. Low-fat dietary intervention and omega-3 supplementation were advised with regular close monitoring. At this stage, a hereditary metabolic liver disease was suspected in the patient with an unexplained liver disease, based on the combination of family history and clinical and laboratory findings.

During the monitoring period, fluctuations in transaminase and triglyceride levels were observed. Transaminase levels remained normal, with some reaching a 1.5–2 fold increase over the upper limit of normal (ULN). Triglyceride levels dropped to 179 mg/dL, but then peaked at 400 mg/dL. During her last clinical evaluation, when she was 12.5 years old, her growth appeared normal, but weight and height for age z-scores decreased slightly compared to baseline, and hepatomegaly (4 cm) persisted. Repeated liver USG showed hepatomegaly and increased echogenicity without evidence of portal hypertension.

### Case-2

A 12-month-old boy with β-thalassemia carrier, who is the sibling of case-1, was referred for evaluation of unexplained liver disease. He was delivered at 37 weeks of gestation, weighing 2800 g. During the neonatal period, he was intubated for respiratory distress and received phototherapy for 1 day due to the jaundice. The mother noticed his abdominal distention when he was 2 months old. Hepatosplenomegaly, elevated transaminases, and hepatosteatosis were detected. The etiology remained unexplained, and close follow-up were recommended.

On admission, his growth was within normal limits. He had pallor, abdominal distension, and marked hepatomegaly (8 cm) and, splenomegaly (5 cm). Laboratory investigation revealed increased levels of liver enzymes, international normalized ratio (INR), triglyceride, and total cholesterol. His hemoglobin and mean corpuscular volume levels were decreased indicating hypochromic microcytic anemia. Abdominal USG showed hepatomegaly, grade 2 hepatosteatosis and splenomegaly. Further biochemical, metabolic and viral investigations were normal except for anti-smooth muscle and anti-liver cytosolic antigen type 1 antibodies positivity and low serum IgA level. A liver biopsy was recommended, but the family refused. Similar to her sister, close monitoring with a low-fat diet and omega-3 supplementation was recommended. At 2 years and 4 months, his height was below the expected average for his age, with a z-score of -2.2, while his weight was also below average, with a z-score of -1.78 at this time. Insulin-like growth factor 1 (IGF-1) was 13 ng/mL (SDS -2.42 the actual age) and insulin-like growth factor binding protein-3 (IGFBP-3) was 1266 ng/mL (SDS -1.04 for the actual age). It was thought that the low IGF-1 level in the patient might be related to chronic liver disease and follow-up was recommended.

During the subsequent years, there was a persistent elevation in liver enzymes, with ALT levels reaching three times the upper reference range limit and GGT levels reaching nine times the upper reference range limit. Over the subsequent years, there was a persistent elevation in liver enzymes, with peak AST levels reaching 15 times the upper limit of normal (x15 ULN), ALT levels reaching 6 times the upper limit of normal (x6 ULN), and GGT levels reaching 4 times the upper limit of normal (x4 ULN). Additionally, while the triglyceride level returned to normal (144 mg/dL), it subsequently increased again to 209 mg/dL. At the latest follow-up when the patient was 4.5 years old, marked hepatosplenomegaly persisted, but growth and psychomotor development were normal. Biochemically, mildly elevated transaminases and triglycerides persisted.

**Fig. 1:**
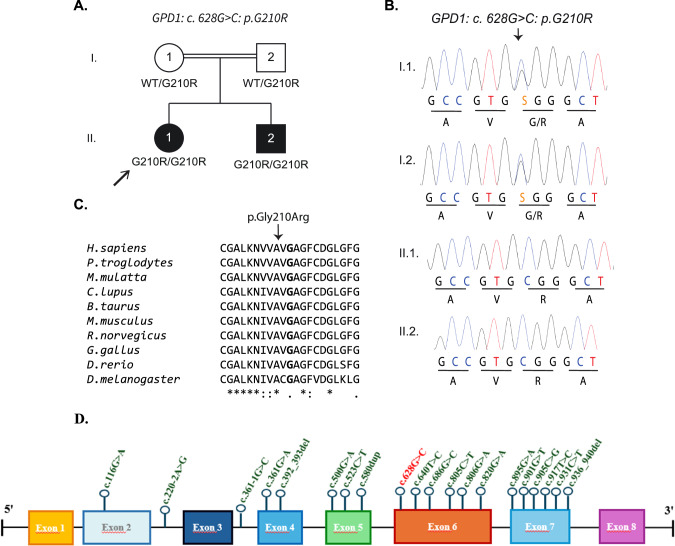
A novel homozygous *GPD1* mutation in two siblings with HTGTI. **A** Pedigree of the family, the patients are shown in black and the healthy individuals are shown in white. Where available *GPD1* mutation (p.G210R) is indicated. **B** Familial segregation of the *GPD1* mutation and its homozygous state in the patient were confirmed by Sanger sequencing. **C** Conservation of the p.G210 residue, highlighted in bold, across various species. (Source: NCBI HomoloGene). **D** Schematic representation of GPD1 mutations. The novel mutation identified in this study are indicated in bold red letters

Clinical and biochemical features and genetic sequencing data of the patients are shown in Tables 1, 2 and Fig. 1. We have reviewed 18 studies, and 45 cases reported in the literature so far. We have compiled the cases and outcomings in the Table. (supplementary Table) The variants described so far in the literature are shown schematically in Fig. 1D [2–19].

**Table 1:** Clinical findings of the patients at presentation and follow-up

	Case 1	Case 2
**Birth characteristics**		
*Mode of delivery*	Cesarean section	Cesarean section
*Birth weight (g)*	4200	2800
*Week of gestation*	42	37
**Age at onset of disease (months)**	18	2
**Symptoms**	Hepatomegaly	Hepatosplenomegaly
**On the admission**		
*Age (years)*	8	1
*Weight (kg), (z score)*	22 (–0.93)	7.7 (–1.36)
*Height (cm), (z score)*	124 (–0.48)	71 (–1.47)
*Liver size (cm)*	3	8
*Spleen size (cm)*	Not palpable	5
**Last clinical evaluation**		
*Age (years)*	12.5	4.5
*Weight (kg) (z score)*	34 (–1.48)	14 (–1.91)
*Height (cm) (z score)*	147 (–0.85)	102 (–1.29)
*Liver size (cm)*	3	10
*Spleen size (cm)*	Not palpable	5

**Table 2:** Biochemical profile of patients with transient infantile hypertriglyceridemia at presentation and follow-up

	Normal values	Patients	At the time of admission	Follow-up				The lowest value during follow-up	The highest value during follow-up
				*Year 1*	*Year 2*	*Year 3*	*Year 4*		
**Fasting blood glucose**	74–100 mg/dL	*Case 1*	76	88	80	81	88	67	108
		*Case 2*	68	93	66	69	65	40	126
**Total bilirubin**	*0.3–1.2 mg/dL*	*Case 1*	0.41	0.81	0.35	0.91	0.38	0.35	1.03
		*Case 2*	1.89	1.21	1.12	1.12	1.52	0.74	2.64
**Direct bilirubin**	0–0.3 mg/dL	*Case 1*	0.07	0.15	0.15	0.28	0.18	0.07	0.33
		*Case 2*	0.65	0.26	0.38	0.39	0.47	0.16	0.8
**Total protein**	60–80 g/L	*Case 1*	70	71.6	73.9	74.2	77.5	67	77.5
		*Case 2*	70	85.6	84.8	76.4	83	66.2	88.5
**Albumin**	35–52 g/L	*Case 1*	44.4	46.4	49.6	50.9	50.7	43.1	54.5
		*Case 2*	37.5	55.8	55.1	52	57.2	35.3	58.5
**AST**	0–50 U/L	*Case 1*	26	28	46	44	62	25	100
		*Case 2*	134	119	91	153	160	86	762
**ALT**	0–50 U/L	*Case 1*	22	27	35	39	45	22	81
		*Case 2*	69	86	61	82	91	40	286
**GGT**	6–42 U/L	*Case 1*	41	40	52	72	70	40	90
		*Case 2*	237	124	66	66	85	64	171
**ALP**	129–417 U/L	*Case 1*	282	302	276	282	448	238	448
		*Case 2*	160	169	146	130	160	122	191
**INR**	0.83–1.17	*Case 1*	1.19	1.14	0.99	1.3	1.18	0.99	1.33
		*Case 2*	1.39	1.22	1.27	1.22	1.29	1.13	1.49
**Triglyceride**	<150 mg/dL	*Case 1*	353	204	223	311	400	179	400
		*Case 2*	379	184	147	161	203	144	379
**Total cholesterol**	<200 mg/dL	*Case 1*	216	195	172	226	166	166	226
		*Case 2*	138	99	143	137	138	95	163
**LDL**	<130 mg/dL	*Case 1*	100	107	89	124	51	51	125
		*Case 2*	54	35	71	64	65	35	79
**HDL**	40–60 mg/dL	*Case 1*	45	47.1	38	40	35	38	52.5
		*Case 2*	8	27.5	43	41	32	8	45.6

The following abbreviations are used: *AST* aspartat aminotransferase, *ALT* alanine aminotransferase, *GGT* gamma-glutamyl transferase, *ALP* alkaline phosphatase, *INR* international normalized ratio, *LDL* low density lipoprotein, *HDL* high density lipoprotein

### Genetic analysis

Whole-exome sequencing (WES) was performed on genomic DNA isolated from the peripheral blood samples of both patients as previously described [20]. First, variants of low quality with reads less than 10 and mapping quality less than 40, and genotype quality less than 30 were excluded. Variants with a higher minor allele frequency of 1% in gnomAD v2.1.1 were eliminated. Only the predicted to be loss-of-function variants (essential splicing, frameshift indels, start-loss, stop-loss, and stop-gain), in-frame indels and missense variants were retained for further analysis. We next focused on the common variants present in both patients and identified a homozygous mutation, NM_005276.4:c.628 G > C:p.G210R, in *GPD1*. This mutation was not listed in any public genome databases. Biallelic variants in *GPD1* have been previously associated with HTGTI. Her parents were heterozygous for the mutation, confirmed by Sanger sequencing, consistent with an autosomal recessive mode of inheritance (Fig. 1A, B). The p.G210R affected an evolutionary conserved Gly residue (Fig. 1C) and was predicted to be damaging by in silico algorithms such as CADD, MutationTaster2021, PolyPhen-2 and SIFT.

## Discussion

HTGTI is a rare cause of childhood transaminase elevation and hepatosteatosis. In 2022, Wang et al. reviewed 31 genetically confirmed patients from 10 case reports published in medical research databases and reported a gender distribution with a ratio of 1.6 males to 1 female. The age of disease onset displayed significant variation between 0 and 7 years. Approximately 40% of the patients had parental consanguinity, and some had a family history of fatty liver [21]. The main characteristics of the disease consist of moderate to severe elevation in triglyceride levels, increased transaminase levels, hepatomegaly, fatty liver, and fibrosis, typically manifesting during early childhood. Splenomegaly, short stature, vomiting, characteristic organic aciduria, intrahepatic cholestasis, elevated bile acids, obesity, and insulin resistance have also been documented [21]. Other rare phenotypes include hypoglycemia, renal disease, and hepatic adenoma [4, 14]. Our patients were clinically similar to other children reported in the literature: they had a family history of consanguineous marriage and a family history of unexplained fatty liver disease and hepatosplenomegaly. They presented with elevated transaminase levels since early infancy, hypertriglyceridemia, marked hepatomegaly and, hepatosteatosis. Although not seen in all patients with HTGTI, growth retardation and/or short stature may be observed in some ( ~ 23%) [2, 3, 11]. One of the sibling (Case2) had short stature accompanied by low IGF-1, which was thought to be related to chronic liver disease, and short stature improved in follow-up.

Elevated transaminases 1.5-3 times higher than normal is a common laboratory finding in patients with HTGTI, but Case 2 had transient, non-recurrent severe enzyme elevation. Similar to other cases in the literature, although transaminases returned to normal in both cases, they were elevated at the last clinical evaluation [2].

GPD1 deficiency is a significant molecular etiology for primary hypertriglyceridemia that initiates during infancy. [2]. It is a laboratory finding seen in approximately 98% of cases [21]. It has been observed that serum triglyceride level reaches the highest values, especially in the first 6 months of life and tends to decrease with age. Although triglyceride levels decreased in Case-1 and returned to normal in Case-2, both had elevated triglyceride levels at the last clinical evaluation. GPD1 mutation in HepG2 cells has been demonstrated to result in elevated triglyceride production and release [2]. Nevertheless, the precise mechanism underlying hypertriglyceridemia in GPD1 deficiency remains elusive and necessitates additional clarification. Like the cases documented in the literature, LDL levels in Case-2 were diminished during early stages and gradually rose to within normal range subsequently [2].

It has been suggested that the fatty liver observed in all patients with GPD1 deficiency on ultrasonography or other imaging may result from excessive acylation of dihydroxyacetone phosphate (DHAP) [3]. The detailed mechanisms remain unclear, necessitating further research for clarification. Hepatomegaly coupled with fatty liver can result from various inherited metabolic liver disorders, such as glycogen storage disease, lipidosis, lysosomal diseases, and citrin deficiency. Genetic analysis and liver biopsy serve as valuable tools in distinguishing GPD1 deficiency from these conditions; nevertheless, the conclusive diagnosis of the disease hinges on genetic testing. Genetic testing was performed on two siblings with a family history of fatty liver disease and unexplained liver disease resulting from consanguineous marriage, considering the possibility of a metabolic hereditary disease. A previously-undescribed homozygous variant, c.628 G > C:p.G210R, in *GPD1* was identified via whole exome sequencing.

The disease’s natural progression remains unclear. While liver fibrosis is typically observed in early infancy among most patients, the prognosis for all affected individuals is favorable, with most experiencing improvements in triglyceride and liver enzyme levels. Some patients with GDP-1 mutations may require treatment as triglyceride levels remain high. Matarazzo et al. reported normalization of triglyceride levels with fenofibrate treatment in a 14.5-year-old boy with severe hypertriglyceridemia, homozygous for the c.895 G > A p.(G299R) variant [7]. Lipoprotein apheresis is not recommended as a routine treatment in these patients, as triglyceride levels may improve without specific therapy, but may be an option for patients with severe hyperlipidemia [6]. Severe hypertriglyceridemia was never observed in the follow-up of our patients.

In the follow-up of children with GPD1 deficiency, growth and development should be evaluated and liver tests, lipid profile, abdominal USG, FibroScan, and other abnormal tests should be checked. Elevated triglyceride levels contribute independently to the risk of coronary artery disease and are additionally associated with heightened susceptibility to acute pancreatitis. The long-term ramifications of hypertriglyceridemia in these patients remain uncertain, underscoring the importance of exercising caution during extended follow-up periods [6].

In conclusion, the rare GDP-1 mutation should be considered in the differential diagnosis of unexplained hepatosteatosis, hypertriglyceridemia, and elevated transaminases. Different variants of the GPD-1 gene should be examined during diagnosis. Although it appears to be a benign disease, more studies are needed to explore further information about the long-term prognosis.

## Supplementary information

Table S1
