# Supplementary material for: GPD1 deficiency—a rare, overlooked cause of liver disease
Source: J Hum Genet. 2025 Apr 11;70(7):375–9. doi: 10.1038/s10038-025-01339-9 (PMC12137118; doi:10.1038/s10038-025-01339-9)
Supplement: Supplementary file 1 — Table S1 [file 10038_2025_1339_MOESM1_ESM.docx]

**Table S1. GPD1 gene variant and phenotype of patients reported worldwide**

| **Case Reports** | **Basel-Vanagaite et al.** | **Joshi et al.** | **Dionisi-Vici et al.** | **Li et al.** | **Li et al.** | **Matarazzo et al.** |
| --- | --- | --- | --- | --- | --- | --- |
| **Year** | 2012 | 2014 | 2016 | 2017 | 2018 | 2020 |
| **Number of cases** | 10 | 1 | 4 | 1 | 1 | 1 |
| **Ethnicity** | Israeli–Arab | Caucasian | Arab-1 Italian-2 NA-1 | Chinese | Chinese | Russian |
| **Age at diagnosis (months)** | 1–9 | 5 | 5–24 | 164 | 3 | 12 |
| **Consanguinity** | 7/10 | NA | 3/4 | No | No | NA |
| **Zygosity** | Homozygous | Compound heterozygous | Homozygous | Compound heterozygous | Homozygous | Homozygous |
| **Nucleotide and amino acid variation** | c.361−1G>C | c.686G>C, p.(Arg229Pro), and a short deletion | Case A: c.806G > A, p.(Arg269Gln)  Case B: c.361-1G > C  Case C & D: c.640T>C, p.(Cys214Arg) | c.220-2A>G and c.820G>A; p.(Ala274Thr) | c.523C>T, p.(Gln175Ter) | c.895G>A, p.(Gly299Arg) |
| **Hepatomegaly** | 10/10 | Yes | 4/4 | NA | Yes | Yes |
| **Splenomegaly** | 3/10 | NA | 1/4 | NA | No | No |
| **Growth Failure** | 4/10 | Yes | 0/4 | Obesity and short stature | No | No |
| **Fatty Liver** | 10/10 | Yes | 4/4 | Yes | Yes | Yes |
| **ALT (U/L)** | 51–230 | 160,2 | 72–179 | Normal | 68-110 | 60 |
| **TG (mg/dL)** | 250–6244 | 838.9 | 170–1180 | Normal | 369–968 | 274 |
| **TC**  **(mg/dL)** | 66–420 | 197.2 | 155-309 | Normal | 91-168 | 166 |
| **Follow-up** | ALT: 4/10 normalized, 6/10 remained elevated.  TG: 1/10 normalized, 7/10 decreased but above 200 mg/dL.  The oldest individual, 23 years of age at the last follow-up, remains asymptomatic, with short stature, elevated transaminases, and a hyperechoic liver compatible with fatty change. | TG level reduced to 536 mg/dL and developmentally, the patient was appropriate for her age at 18 months old | Patient A: At the last follow-up, the patient was 4.5 years old and had normal TG and almost normal ALT, but the liver was enlarged and fatty infiltration continued  Patient B: TG level normalized at the age of 4 years  Patient C: At the last control at the age of 7, hepatomegaly continued, TG was 271 mg/dl, ALT was 49 IU/L.  Patient D: Mild hepatomegaly and hypertriglyceridemia (358–407 mg/dL) are still present at 31 years old | NA | At the last 15-month follow-up, hepatomegaly, hypertriglyceridemia, and moderately elevated transaminases were present | At the age of 15, TG increased to 1017 mg/dL. After 15 months of fenofibrate treatment, TG decreased to 147 mg/dL, ALT was normal, and hepatomegaly was present |

**Table 1. Continued**

| **Case Reports** | **Wang et al.** | **Xie et al.** | **Tesarova et al** | **Lin et al.** | **Kumar and Sharma** | **Pengfei et al.** | **Kumar et al.** | **Polchar et al.** |
| --- | --- | --- | --- | --- | --- | --- | --- | --- |
| **Year** | 2021 | 2021 | 2021 | 2021 | 2021 | 2021 | 2022 | 2022 |
| **Number of cases** | 1 | 2 | 10 | 1 | 1 | 1 | 1 | 1 |
| **Ethnicity** | Chinese | Chinese | Romanian-9 Palestinian-1 | Chinese | Indian | NA | Indian | South Asian |
| **Age at diagnosis (months)** | 2 | 5-13 | NA | 4 | 5 | 1 | 7 | 23 |
| **Consanguinity** | No | No | NA | No | No | No | No | Yes |
| **Zygosity** | Compound heterozygous | Compound heterozygous | Homozygous | Homozygous | Homozygous | Compound heterozygous | Homozygous | Homozygous |
| **Nucleotide and amino acid variation** | c.901G>T, p.(Glu301Ter), and short deficiency | c.931C > T and c.901G > T | -9/10 of cases: c.895G>A, p.(Gly299Arg)  -1/10 of cases: c.116G>A, p.(Trp39Ter) | c.523C>T , p.(Gln175Ter) | c.500G>A, p.(Gly167Asp) | c.901G > T, (p.E301X) and c.220-2A>G | c.500G>A, (p.Gly167Asp) | c.500G>A, (p.Gly167Asp) |
| **Hepatomegaly** | Yes | 2/2 | 9/10 | Yes | Yes | Yes | Yes | Yes |
| **Splenomegaly** | Yes | No | NA | No | Yes | No | No | Yes |
| **Growth Failure** | No | No | No | No | NA | No | Yes | Yes |
| **Fatty Liver** | Yes | Yes | NA | Yes | Yes | Yes | Yes | Yes |
| **ALT U/L** | 106 | Elevated | 35–151 | 107 | Normal | 106 | 126 | 98 |
| **TG (mg/dL)** | 566 | Elevated | 189-1062 | 388.5 | Elevated | 1688 | 311 | 787 |
| **TC**  **mg/dL** | 131 | NA | NA | Normal | Normal | 97.4 | 118 | NA |
| **Follow-up (months)** | At 16 months of age, there was no hepatosplenomegaly and TG was normal but elevated transaminases and hepatic steatosis persisted | After treatment with a low-fat diet enriched with medium-chain fatty acids, TG levels decreased significantly, and in 1 patient TG normalized | NA | At the age of 3.5, ALT and TG were normal, liver size was reduced compared to before | At 6-month follow-up, hepatomegaly persisted and TG levels decreased | At 1 year of age, the liver was palpable at 3.2 cm below the costal margin and 3.6 cm below the xiphoid, and ALT 69 U/L, TG 372 mg/dL | At the age of 7, ALT 98 U/L, TG 298 mg/dL and ultrasound revealed non-vascular in appearance suggestive of possible adenoma | At age 7, ALT, TG and hepatomegaly spontaneously improved, developmental delay and weight gain began to improve |

**Table 1. Continued**

| **Case Reports** | **Tan et al.** | **Gunes et al.** | **Sharma et al.** | **Agrawal et al.** | **Present Cases** |
| --- | --- | --- | --- | --- | --- |
| **Year** | 2022 | 2023 | 2024 | 2025 | 2025 |
| **Number of cases** | 1 | 1 | 2 | 5 | 2 |
| **Ethnicity** | Chinese | Turkish | North India | North India | Turkish |
| **Age at diagnosis (months)** | 10 | 3 | 3-15 | 6, 12, 18, 24, and 12 | 96-144 |
| **Consanguinity** | Yes | Yes | No | Yes | Yes |
| **Zygosity** | Homozygous | Homozygous | Homozygous | Case 1&3: compound heterozygous, Case 2,4&5: homozygous | Homozygous |
| **Nucleotide and amino acid variation** | c.805C>T, p.Arg269Trp | c.936_940del (p.His312GlnfsTer24) | c.580dup, p.(Val194GlyfsTer22) | Case1: c.392_393del, p.(Leu131HisfsTer27), and c.500G>A, p.(Gly167Asp)  Case2: c.917T>C, p.(Leu306Pro)  Case3: : c.361G>A, p.Gly121Arg), and c.500G>A, p.(Gly167Asp)  Case4: c.905C>G, p.(Thr302Arg)  Case5: c.500G>A, p.(Gly167Asp) | c.628G>C, p.(Gly210Arg) |
| **Hepatomegaly** | Yes | Yes | 2/2 | 5/5 | 2/2 |
| **Splenomegaly** | No | No | 2/2 | 4/5 | 1/2 |
| **Growth Failure** | No | Yes | 1/2 | 2/5 | 1/2 |
| **Fatty Liver** | Yes | Yes | 2/2 | 5/5 | 2/2 |
| **ALT (U/L)** | 46 | 16 | 84-183 | 47–183 | 22-69 |
| **TG (mg/dL)** | 361 | 1603 | 328–353 | 196–989 | 353-379 |
| **TC**  **(mg/dL)** | 88.6 | 209 | 119–179 | 111–128 | 138-216 |
| **Follow-up (months)** | NA | Erythrocyte suspension was needed every 3-4 weeks in the first 3 months. At 18 months of age, TG 442 mg/dL ALT was normal, liver size was at the upper limit of normal but splenomegaly was present | At the end of 1-year follow-up, ALT decreased to 50-87 U/L and TG decreased to 210-220 mg/dL. Hepatomegaly persisted | TG decreased significantly in all patients, ALT normalized in 1 patient. Organomegaly persisted in all patients. To Case-1  fenofibrate and allopurinol initiated | At the end of 4 years of follow-up, TG was 400-203 mg/dL, ALT was 91 U/L in case 2. Hepatomegaly continued in both cases, while splenomegaly persisted in case 2 |

The following abbreviations are used: ALT, Alanine aminotransferase; TG, triglyceride; TC, total cholesterol; NA, not available
